# Crystal Structure of Reovirus Attachment Protein σ1 in Complex with Sialylated Oligosaccharides

**DOI:** 10.1371/journal.ppat.1002166

**Published:** 2011-08-04

**Authors:** Dirk M. Reiter, Johnna M. Frierson, Elizabeth E. Halvorson, Takeshi Kobayashi, Terence S. Dermody, Thilo Stehle

**Affiliations:** 1 Interfaculty Institute of Biochemistry, University of Tuebingen, Tuebingen, Germany; 2 Departments of Pathology, Microbiology, and Immunology, Vanderbilt University School of Medicine, Nashville, Tennessee, United States of America; 3 Elizabeth B. Lamb Center for Pediatric Research, Vanderbilt University School of Medicine, Nashville, Tennessee, United States of America; 4 Department of Pediatrics, Vanderbilt University School of Medicine, Nashville, Tennessee, United States of America; Institut Pasteur, France

## Abstract

Many viruses attach to target cells by binding to cell-surface glycans. To gain a better understanding of strategies used by viruses to engage carbohydrate receptors, we determined the crystal structures of reovirus attachment protein σ1 in complex with α-2,3-sialyllactose, α-2,6-sialyllactose, and α-2,8-di-siallylactose. All three oligosaccharides terminate in sialic acid, which serves as a receptor for the reovirus serotype studied here. The overall structure of σ1 resembles an elongated, filamentous trimer. It contains a globular head featuring a compact β-barrel, and a fibrous extension formed by seven repeating units of a triple β-spiral that is interrupted near its midpoint by a short α -helical coiled coil. The carbohydrate-binding site is located between β-spiral repeats two and three, distal from the head. In all three complexes, the terminal sialic acid forms almost all of the contacts with σ1 in an identical manner, while the remaining components of the oligosaccharides make little or no contacts. We used this structural information to guide mutagenesis studies to identify residues in σ1 that functionally engage sialic acid by assessing hemagglutination capacity and growth in murine erythroleukemia cells, which require sialic acid binding for productive infection. Our studies using σ1 mutant viruses reveal that residues 198, 202, 203, 204, and 205 are required for functional binding to sialic acid by reovirus. These findings provide insight into mechanisms of reovirus attachment to cell-surface glycans and contribute to an understanding of carbohydrate binding by viruses. They also establish a filamentous, trimeric carbohydrate-binding module that could potentially be used to endow other trimeric proteins with carbohydrate-binding properties.

## Author Summary

Human reoviruses bind first with low affinity to a carbohydrate receptor that brings the virus in close proximity to the host cell. This interaction then facilitates high-affinity binding to a second receptor, the tight junction component junctional adhesion molecule-A (JAM-A). While all human reoviruses bind JAM-A, they differ in carbohydrate receptor specificity, and this difference may influence the distinct disease patterns of reovirus serotypes. We present here the structure of the attachment protein of type 3 reovirus in complex with carbohydrates that naturally occur on human cells. Our results show that the protein forms an elongated trimer, with the carbohydrate binding site being located close to the midpoint of the molecule in a fiber-like region. Our findings provide insights into mechanisms of reovirus attachment to cell-surface glycans and contribute to an understanding of carbohydrate binding by viruses. They also establish a filamentous, trimeric carbohydrate-binding module that could potentially be used to introduce carbohydrate-binding properties into other trimeric proteins.

## Introduction

Viral infections are initiated by specific attachment of a virus particle to receptors at the surface of the host cell. This process, which serves to firmly adhere the virus to its cellular target, is rarely a bimolecular interaction between one viral attachment protein and one receptor. In most cases, several receptors are employed, and recognition events are frequently accompanied by substantial structural rearrangements that serve to expose new binding sites, strengthen the initial interaction, and prime the virus for cell entry. Structure-function analyses of virus-receptor interactions have provided detailed insights into the attachment strategies of viruses belonging to several different families [1]–[18]. However, much less is known about structure-function interrelationships between different binding sites for distinct receptors on the same viral attachment molecule.

Reoviruses are useful experimental models for studies of virus-receptor interactions and viral pathogenesis. Moreover, the recent development of plasmid-based reverse genetics for reovirus provides an opportunity to manipulate these viruses for oncolytic and vaccine applications. Reoviruses form icosahedral particles approximately 850 Å in diameter. At the virion five-fold symmetry axes, the trimeric attachment protein, σ1, extends from pentameric turrets formed by the λ2 protein. A similar arrangement of a trimeric attachment protein inserted into a pentameric base is also observed for the adenovirus attachment protein, fiber. The σ1 protein is about 400 Å long and consists of three discrete domains, termed tail, body, and head [19]. Residues 1 to 160 encompass the tail domain, which partially inserts into the virion capsid [20]–[22]. This region of the molecule is predicted to form an α-helical coiled-coil structure. The body domain encompasses residues 170 to 309 and contains β-spiral repeat motifs [22]. Lastly, the globular head domain incorporates residues 310 to 455 and folds into an 8-stranded β-barrel [22], [23].

Reovirus attachment is thought to proceed via a two-step adhesion-strengthening mechanism, in which σ1 first engages widely distributed carbohydrate receptors with lower affinity. The three prototype reovirus strains, type 1 Lang (T1L), type 2 Jones (T2J), and type 3 Dearing (T3D) recognize different carbohydrate structures, which may account for the serotype-specific differences in routes of spread in the host and end-organ tropism. In the case of serotype 3 (T3) reoviruses, the carbohydrate bound is α-linked sialic acid [24]–[26]. This initial contact, which has lower affinity and may allow for lateral diffusion of the particle at the membrane [27], is followed by high-affinity interactions with junctional adhesion molecule-A (JAM-A) [28], a component of tight junctions [29]–[31]. All reoviruses, including prototype and field-isolate strains, use JAM-A as a high-affinity receptor [28], [32], [33]. Firm adherence to the cell triggers uptake of the particle, which is dependent on β1 integrins [34], [35].

Discrete regions of σ1 mediate binding to its cell-surface receptors. Structural and functional analyses show that the σ1 head, which projects farthest from the virus capsid, engages JAM-A [33], [36], [37]. In contrast, sequences in the σ1 body bind to carbohydrates [38]. Sequence analysis of reovirus variants identified three residues, Asn198, Arg202, and Pro204, as likely critical for the interaction of T3 σ1 with sialic acid. These residues lie near the midpoint of the protein, at the lower end of the body domain, about 100 Å away from residues in the head that interact with JAM-A. Earlier structural analyses of T3D σ1 [22], [23], [36] were based on constructs that did not include this putative carbohydrate-binding site. It is therefore currently unclear how σ1 achieves its specificity for sialic acid, whether the large distance between the two receptor-binding sites on σ1 is relevant for binding, or whether σ1 undergoes rearrangements after engaging its carbohydrate receptor.

To enhance an understanding of mechanisms by which viral attachment proteins engage cell-surface glycans, we determined the crystal structure of T3D σ1 in complex with α-2,3-sialyllactose, α-2,6-sialyllactose, and α-2,8-disiallylactose. All three carbohydrates terminate in sialic acid but feature different linkages that are present in various physiologic glycans. In addition, we used plasmid-based reverse genetics to engineer reoviruses that express mutagenized forms of σ1 to define residues required for functional binding to sialic acid. These studies shed light on the structural basis of σ1-sialic acid interactions and define a new carbohydrate-binding structural motif in a viral attachment protein.

## Results

### Construct Design and Structure Determination

The σ1 protein belongs to a class of fiber proteins constructed from triple β-spirals, a motif that was first identified in the adenovirus fiber [39]. In a previous study, we crystallized a smaller region of σ1, spanning residues 246 to 455 and containing three β-spiral repeats as well as the globular head domain [22]. While this structure provided no insights into the carbohydrate-binding region of σ1, it served as a basis to predict that β-spiral repeats form the entire body domain of the protein (residues 167–309) [22]. Near residue 170, the body domain transitions into a long α-helical coiled-coil region that forms the N-terminal tail domain (residues 1–156).

To determine the structure of a longer fragment of σ1 including the predicted sialic-acid binding residues 198, 202, and 204, we designed a construct for the expression of residues 170–455. This construct excluded the long α-helical coiled-coil region to simplify protein expression, purification, and crystallization. Prototype strain T3D σ1 is sensitive to trypsin-mediated cleavage after Arg245 [40]. However, a sequence polymorphism occurring in the majority of T3 field-isolate strains, Thr249Ile, renders the protein resistant to trypsin [40]. A construct containing Ile249 was therefore used in our study. Trimerization was promoted by using a hexahistidine-tagged trimerization domain, a modified GCN4 sequence [41], at the N-terminus of the expressed protein. This domain was proteolytically removed before final purification and crystallization.

### Overall Structure of σ1

**Figure 1:**
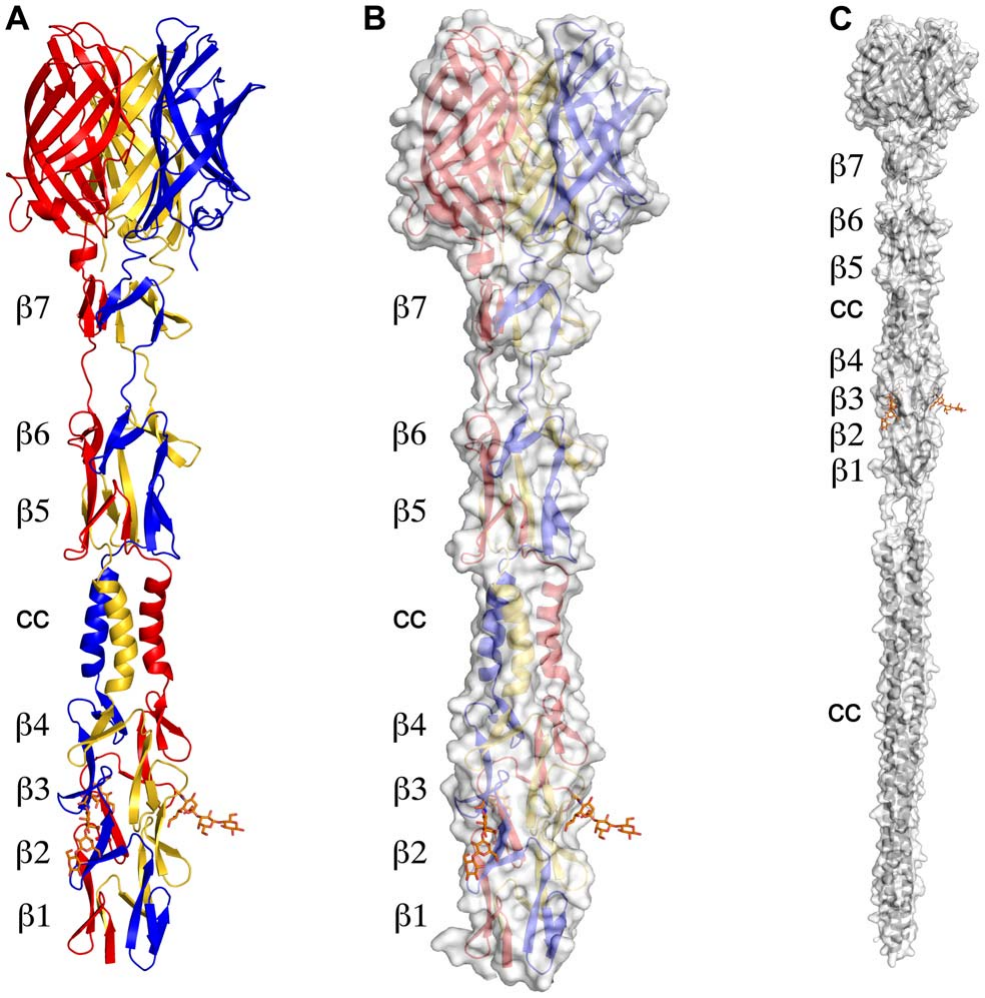
Structure of T3D σ1. (**A**) Ribbon drawing of the T3D σ1 body and head domains in complex with α-2,3-sialyllactose. The σ1 monomers are shown in red, blue, and yellow. The body domain consists of seven triple β-spiral repeats (β1–β7) and an α-helical coiled-coil domain (cc) that is inserted between β-spiral repeats β4 and β5. The bound α-2,3-sialyllactose is shown in stick representation and colored in orange. (**B**) Molecular surface of the σ1 structure, shown in semitransparent white coloring. (**C**) Model of full-length σ1, including a computer-generated trimeric α-helical coiled coil structure spanning σ1 residues 1–160 at the N-terminus.

**Figure 2:**
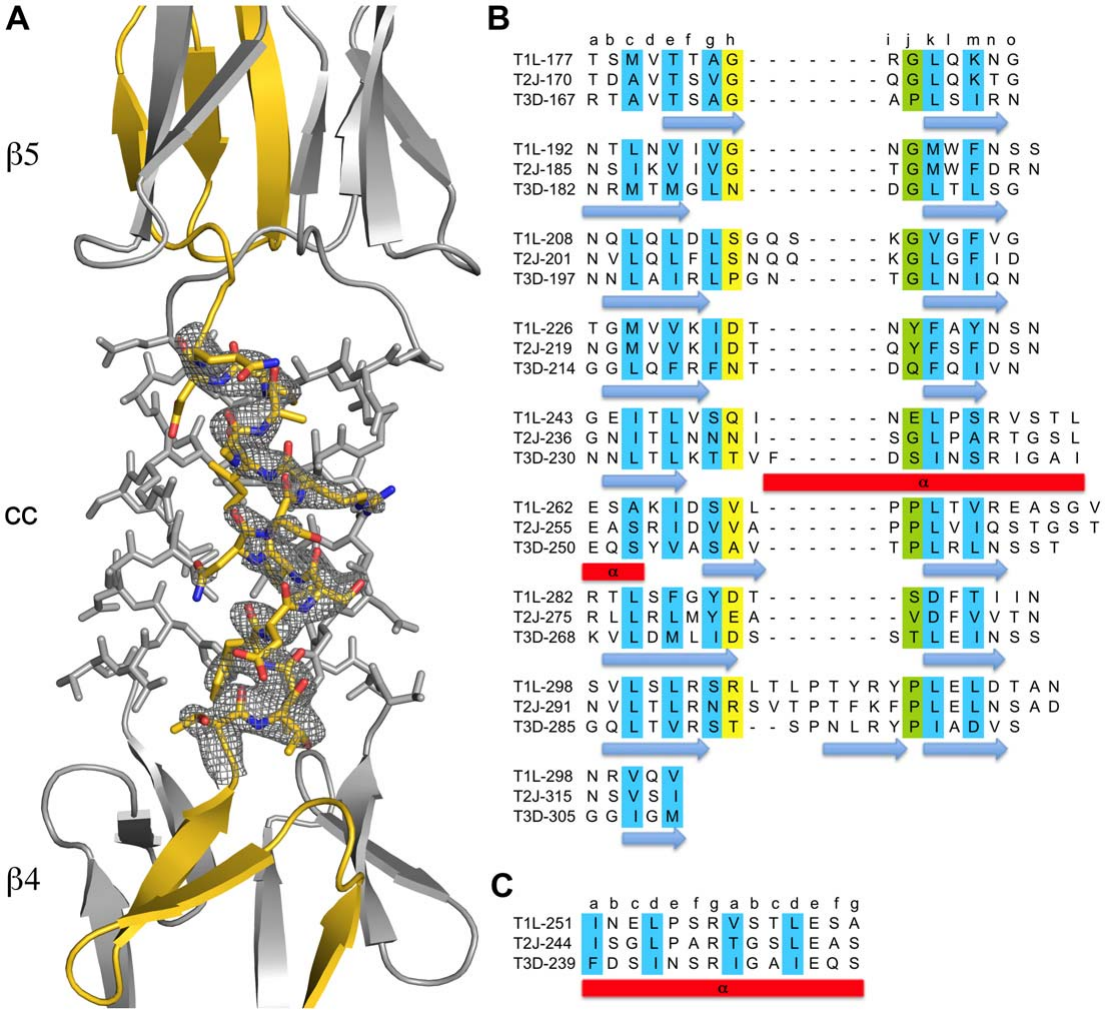
Structural features of the T3D σ1 body domain and sequence alignments with T1L and T2J σ1. (**A**) Close-up view of the α-helical coiled-coil that separates β-spirals 4 and 5 in the T3D σ1 body domain. The coiled-coil region is shown as a stick model, while the adjacent β-spiral repeats are depicted as a ribbon drawing. A simulated annealing omit difference map for one chain is shown with a radius of 2.2 Å and a contour level of 3σ. (**B**) The residues are aligned according to the triple β-spiral consensus sequence (a–o), with typically hydrophobic residues (c, e, g, k, and m) indicated in cyan and the position in the β-turn usually occupied by proline or glycine (j) in green. (**C**) Sequence analysis of the coiled-coil region in the body domain. Residues 239–252 are organized in heptads (a–g), and the coiled-coil consensus is indicated with typically hydrophobic residues (a and d) highlighted in cyan.

The structure of σ1 residues 170 to 455 reveals a highly elongated, symmetric trimer that measures about 200 Å in length (Table 1 and Figure 1A,B). Tail residues N-terminal to amino acid 170, which were not included in the crystallized protein, are predicted to form an α-helical coiled-coil structure that adds another 200 Å in length to the protein (Figure 1C). As expected, the structure of the globular head domain (residues 310 to 455) is essentially identical to that described previously [22]. However, the body domain displays a number of unusual features. Although sequence-based predictions suggested that this region would be composed of eight consecutive triple β-spiral repeats [22], we find that the body domain contains a mixture of α-helical coiled-coil and β-spiral repeats (Figure 1). Four β-spiral repeats at the N-terminus (β1–β4, residues 170 to 235) are followed by a short α-helical coiled-coil (cc, residues 236 to 251) and three additional β-spiral repeats (β5-β7, residues 252 to 309) (Figure 2). Inspection of the sequence indicates a likely reason for the deviation from the β-spiral fold at the center of the body (Figure 2B). Three hydrophilic residues (Thr236, Ser244, and Ser252) are located at positions that are typically occupied by hydrophobic side chains in β-spirals. Moreover, Ser241 replaces a characteristic proline or glycine at the turn in a β-spiral repeat. While some deviations from the β-spiral consensus sequence can be tolerated, even residues replacing the glycine or proline (e.g., residues Gln224 or Thr278), the cumulative effect of the four non-consensus residues results in a β-spiral no longer being the optimal fold. The α-helical coiled-coil structure contains two heptad-repeat sequences, starting with Phe239 and ending with Gln251 (Figure 2A,C).

**Table 1:** Data collection and refinement statistics.

σ1 in complex with	α-2,3-sl	α-2,6-sl	α-2,8-di-sl
Space group	P2_1_2_1_2	P2_1_2_1_2	P2_1_2_1_2
Unit cell dimensions (Å)	a = 87.15	a = 87.61	a = 87.19
	b = 333.18	b = 333.06	b = 331.84
	c = 58.49	c = 58.29	c = 58.13
Unit cell angles (°)	α = β = γ = 90	α = β = γ = 90	α = β = γ = 90
Resolution range (Å)	38.6–2.25	48.05–2.79	48.06–2.28
Completeness (%)	95.37	98.65	96.03
Total reflections	369038	547842	277926
Unique reflections	78324	43203	75913
R_merge_ (%)#	10.8	6.2	9.6
I/σI	13.5	18.6	18.6
R_work_ (%)*	15.77	15.69	17.31
R_free_ (%)*	19.89	20.48	22.03
r.m.s.d. bond lengths (Å)	0.006	0.007	0.006
r.m.s.d. bond angles (°)	0.960	1.12	1.01

r.m.s.d., root-mean-square deviation. sl, sialyllactose.

*R_work_  =  R_free_  =  Σ| |F_obs_(hkl) | - |F_calc_ (hkl) | |/Σ |F_obs_ (hkl). R_free_ was calculated with 5% of the data.

# R_merge_  =  Σ | *I* - <*I*> |/Σ*I*

### Structure of σ1 in Complex with α-2,3-Sialyllactose

**Figure 3:**
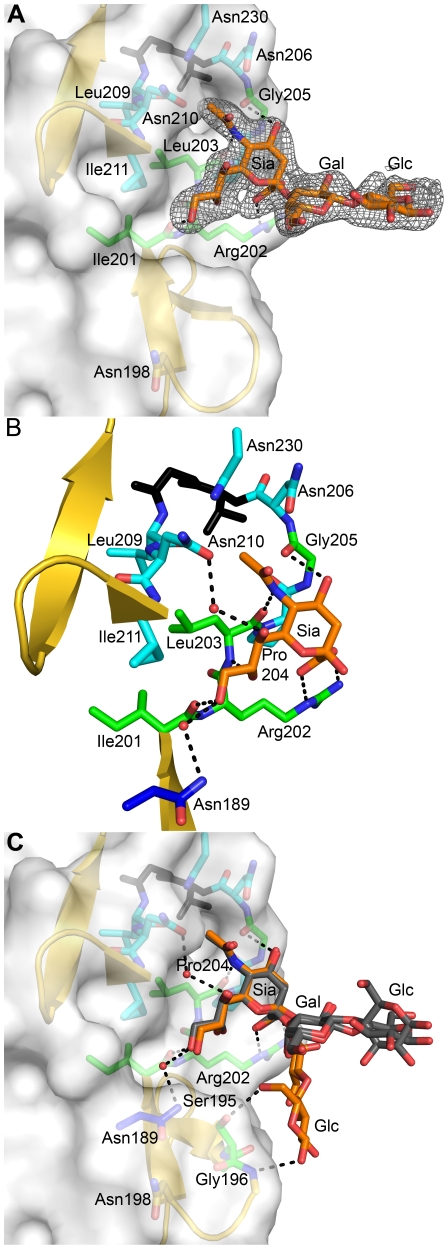
Interactions between σ1 and sialic acid. (**A**) Simulated annealing omit difference density map contoured at 3σ and displayed with a radius of 2.2 Å around the bound α-2,3-sialyllactose. The sugar moieties are labeled Sia (sialic acid), Gal (galactose), and Glc (glucose) here and in subsequent figures. (**B**) Detailed interactions between σ1 and the terminal sialic acid of α-2,3-sialyllactose. Residues in the binding region are drawn in ball and stick representation, while the rest of the protein is shown as a ribbon drawing. The σ1 residues forming hydrogen bonds or salt bridges with the ligand are shown in green, and residues forming van der Waals contacts are shown in cyan. The side chain of Asn189 (colored dark blue) is contributed by a neighboring σ1 monomer (see also Figure 1A). Sialic acid is shown in ball-and-stick representation, with carbons colored orange, oxygens colored red, and nitrogens colored blue. Bridging waters are shown as orange spheres. Hydrogen bonds and salt bridges are represented with broken lines. (**C**) Superposition of all three bound ligands into a single binding site. The superposition was performed using σ1 residues only. While the orientation of the terminal sialic acid is nearly identical, the lactose moieties are facing in different orientations as a result of their participation in different crystal contacts.

To elucidate the structural basis of the interaction of the reovirus attachment protein σ1 with its carbohydrate coreceptor, we prepared a complex by soaking crystals of σ1 with 10 mM α-2,3-sialyllactose, a compound that terminates in α-linked sialic acid. The subsequent structure, determined at 2.25 Å resolution (Table 1), unambiguously demonstrated the location of the carbohydrate in an unbiased difference electron-density map (Figure 3A). The oligosaccharide binds in a shallow groove next to the loop connecting the second and third β-spiral repeats. The σ1 protein contains three identical binding sites, one on each chain, and all three are occupied by α-2,3-sialyllactose molecules, with the sialic acid making identical and extensive contacts in each chain (Figure 3B,C). The lactose moieties face different directions, probably as a result of internal flexibility and participation in crystal contacts (Figure 3C).

Sialic acid contains four characteristic functional groups: a carboxylate at C1, a hydroxyl group at C4, an N-acetyl group at C5, and a glycerol chain at C6. All four groups are recognized by σ1 (Figure 3B). Arg202 forms a bidentate salt bridge with the carboxyl group. A single hydrogen bond links the hydroxyl group at C4 to the carbonyl of Gly205. The amide of the N-acetyl group is engaged in a hydrogen bond with the backbone carbonyl of Leu203, and the N-acetyl methyl group is facing into a partially hydrophobic cavity. The glycerol chain lies parallel to the peptide backbone, forming direct hydrogen bonds with the backbone carbonyl of Ile201 and the amide nitrogen of Leu203 and in some of the binding sites water-mediated hydrogen bonds with the Asn210 side chain and the amide nitrogen of Ile211. We note that Arg202, which was previously shown to influence sialic acid binding [42], provides a key contact to the ligand. Moreover, Pro204, which also had been implicated in sialic acid binding [42], is part of a structure that shapes the ligand-binding site.

### Structures of σ1 in Complex with α-2,6-Sialyllactose and α-2,8-Disialyllactose

**Figure 4:**
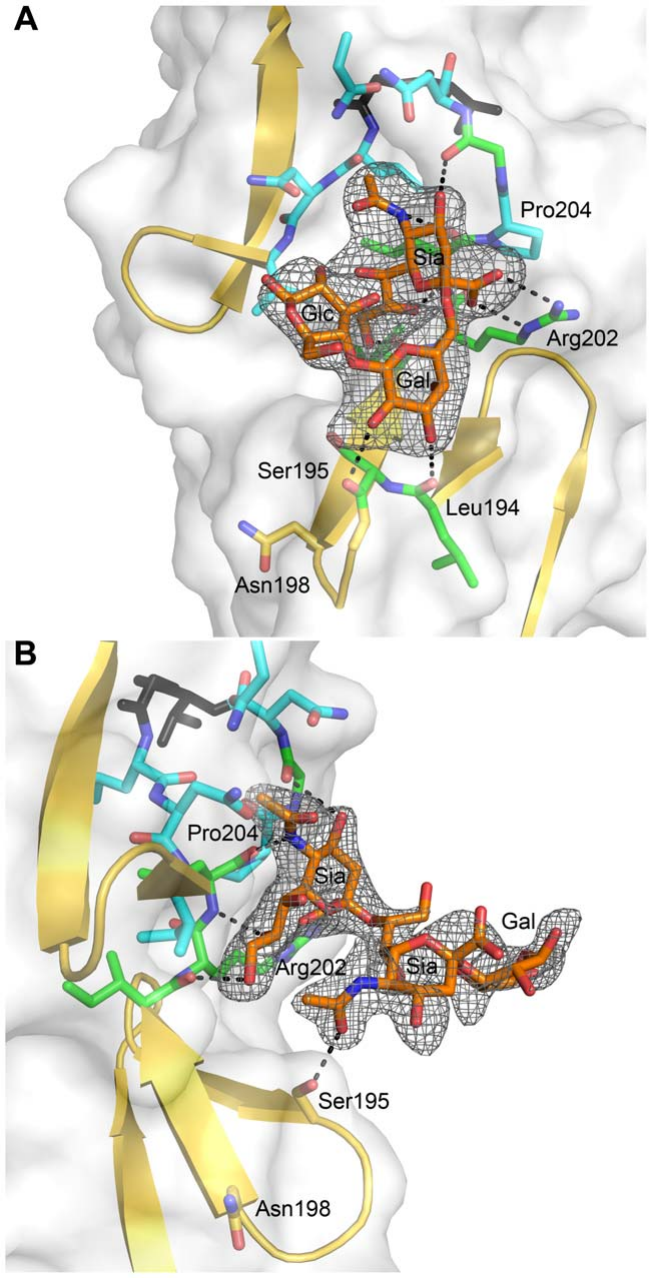
σ1 in complex with sialic acid in different linkages. (A) View into the carbohydrate-binding site of the complex of σ1 with α-2,6-sialyllactose. (B) View into the carbohydrate-binding site of the complex of σ1 with α-2,8-disialyllactose. The orientation in panel (A) differs by 60 degrees along a vertical axis from the orientations shown in panel (B) and Figure 3 to provide a clearer view into of the binding site. In both panels, σ1 residues directly contacting the ligand are shown in green, and surrounding residues making van der Waals contacts are shown in cyan. The ligands are shown in ball-and-stick representation, with carbons colored orange, oxygens colored red, and nitrogens colored blue. Hydrogen bonds are represented with broken black lines. The maps are simulated annealing omit difference density maps contoured at 3σ and displayed with a radius of 2.2 Å around the ligands.

As contacts in the complex of σ1 with α-2,3-sialyllactose exclusively involve the sialic acid moiety, we hypothesized that σ1 should be capable of binding sialic acid in different naturally occurring linkages, including α-2,6- and α-2,8-linked sialic acid. We therefore determined crystal structures of σ1 in complex with α-2,6-sialyllactose (Figure 4A) and α-2,8-disialyllactose (Figure 4B). Refinement statistics for both structures are provided in Table 1. In each case, only two of the binding sites are occupied, as the third is partially blocked by crystal contacts. For the α-2,6-sialyllactose complex, the electron density allowed us to unambiguously identify all three sugar residues (Figure 4A). The electron density for the α-2,8-disialyllactose complex did not allow us to model the terminal glucose. Comparison of these structures with each other and with the α-2,3-sialyllactose complex shows that the terminal sialic acid is bound in the same conformation and with identical contacts in all three cases. However, the remaining moieties of the glycans differ in conformation and contacts with σ1. The α-2,3-sialyllactose and α-2,8-disialyllactose ligands assume an elongated shape in which the lactose groups face away from the protein (Figure 3C, Figure 4B). Inspection of the α-2,8-disialyllactose complex shows that the N-acetyl group of the second sialic acid forms a hydrogen bond to the side chain of Ser195. In contrast, σ1 binds α-2,6-sialyllactose in a folded-back conformation (Figure 4A). This conformation is stabilized by an intramolecular hydrogen bond and the galactose O2 and O3 hydroxyl groups, which form hydrogen bonds to the backbone carbonyl atoms of Ser195 and Leu194, respectively.

### Residues in Reovirus σ1 Required for Sialic Acid Binding

To identify sequences that influence sialic acid binding, we used plasmid-based reverse genetics [43], [44] to introduce point mutations into the σ1 protein of reovirus strain T3D. Mutant viruses were isolated following co-transfection of murine L929 cells with RNA-encoding plasmids corresponding to the T3D *L1-L3*, *M1-M3*, and *S2-S4* genes and a plasmid corresponding to the σ1-encoding *S1* gene incorporating site-specific mutations. Thus, each recombinant virus is isogenic, with the exception of the *S1* gene and its protein product, σ1. Guided by the structure of the σ1-sialic acid complexes, we engineered individual alanine substitutions of amino acids ranging from Asn189 to Asn210. By their location in the structure, we hypothesized that these residues would be required for functional sialic acid binding. In addition, substitutions N198D, R202W, and P204L, which have been implicated in sialic acid binding by sequence comparisons of reovirus strains that differ in sialic acid utilization [26], [45] and genetic analysis of reovirus mutants adapted to growth in murine erythroleukemia (MEL) cells [42], were engineered to define the effect of these polymorphisms in an otherwise isogenic background.

**Figure 5:**
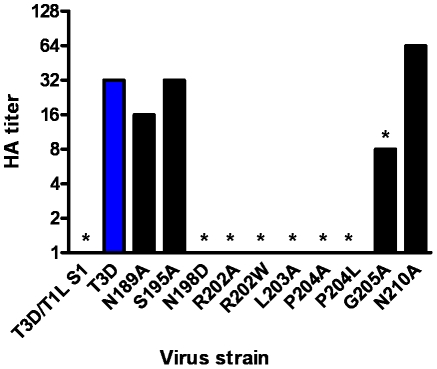
HA assay of T3D σ1 point mutants. Purified reovirus virions (10^11^ particles) were serially diluted in 0.05 ml of PBS in 96-well U-bottom microtiter plates. Bovine erythrocytes were washed twice with PBS and resuspended at a concentration of 1% (vol/vol) in PBS. Erythrocytes (0.05 ml) were added to wells containing virus and incubated at 4°C for at least 2 h. HA titer is expressed as 10^11^ particles divided by the number of particles/HA unit. One HA unit equals the number of particles sufficient to produce HA. *, *P*<0.05 in comparison to T3D (Student's *t* test).

**Figure 6:**
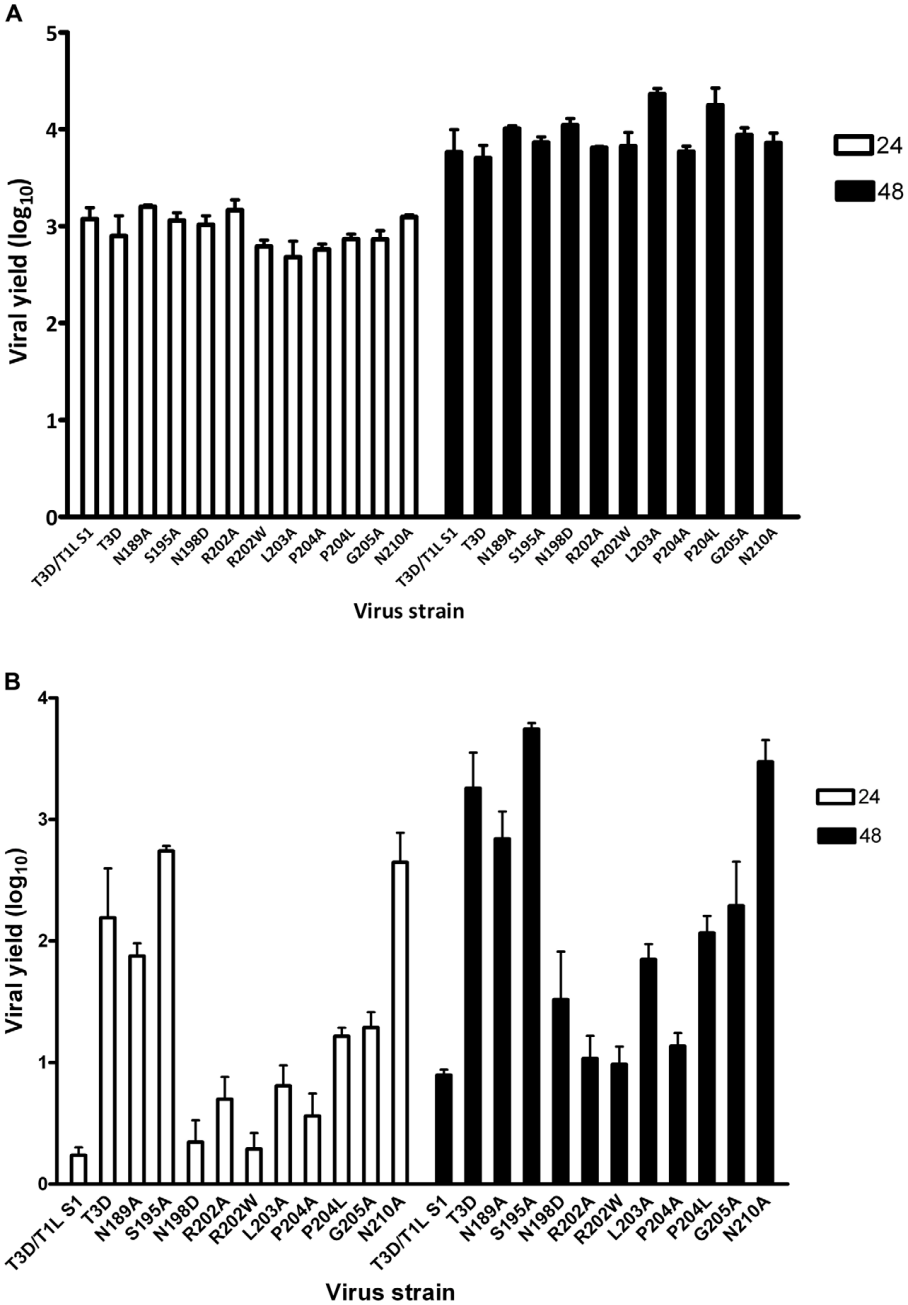
Identification of σ1 residues required for binding to cell-surface sialic acid. **(A)** Infection of murine fibroblast (L929) cells or **(B)** murine erythroleukemia (MEL) cells by wild-type or point-mutant viruses. Cells were adsorbed with virus at an MOI of 1 PFU/cell. Following incubation at room temperature for 1 h, the inoculum was removed, and cells were incubated at 37°C for 24 and 48 h. Viral titers were determined by plaque assay. The results are expressed as viral yields (log_10_ titer at *t* = 24 or 48 h minus log_10_ titer at *t* = 0 h) for triplicate samples. Error bars indicate S.D. *, *P*<0.05 in comparison to T3D (Student's *t* test).

After confirming the σ1-encoding S1 gene nucleotide sequences, the mutant viruses were tested for hemagglutination (HA) capacity (Figure 5) and growth in L929 cells and MEL cells (Figure 6). In comparison to rsT3D, rsT3D-σ1N198D, rsT3D-σ1R202A, rsT3D-σ1R202W, rsT3D-σ1L203A, rsT3D-σ1P204A, rsT3D-σ1P204L, and rsT3D-σ1G205A produced little or no agglutination of calf erythrocytes, a sensitive assay for sialic acid binding [26]. However, rsT3D-σ1N189A, rsT3D-σ1S195A, and rsT3D-σ1N210A produced HA titers that were comparable to those of wild-type rsT3D. Each of the point-mutant viruses produced approximately 1000-fold yields of viral progeny after growth in L929 cells (Figure 6), a cell line that does not require sialic acid binding for reovirus to replicate [45]. In contrast, those containing mutations N198D, R202A, R202W, L203A, P204A, P204L, and G205A displayed attenuated growth in MEL cells (Figure 6), a cell line permissive only to sialic acid binding reovirus strains [45]. These findings indicate that viruses with mutations of residues 198, 202, 203, 204, and 205 are altered in sialic acid binding efficiency, suggesting that these residues serve a functional role in T3D σ1-sialic acid interactions.

## Discussion

Although all known reovirus strains engage cells by binding to the tight junction protein JAM-A [33], the major reovirus serotypes differ in the routes of dissemination in the host and tropism for host tissues [46]–[48]. These differences are linked to the σ1-encoding S1 gene segment and most likely attributable to serotype-specific interactions of σ1 with different cell-surface receptors. T3 reoviruses require sialic acid as a coreceptor, but the context in which sialic acid is bound is unknown. To define this interaction, we determined crystal structures of reovirus σ1 in complex with three sialylated glycans that incorporate a terminal sialic acid moiety in different linkages. These structural analyses were complemented with mutagenesis experiments that establish the physiologic relevance of the observed interactions.

The σ1 protein uses a complex network of contacts to engage terminal sialic acid, which is a common feature of all three glycans studied here. The interactions involve σ1 residues at the lower end of the body domain, between β-spirals 2 and 3. At this location, the sialic acid moiety docks into a shallow pocket that is formed mainly by residues in the third β-spiral. All four functional groups of sialic acid make contacts with σ1 through an elaborate network of hydrogen bonds and van der Waals interactions. Mutations that alter these contacts lead to significantly reduced sialic acid binding as assessed by HA profiles and diminished infection of MEL cells. Although all three ligands used for complex formation with σ1 contain additional carbohydrates, these make very few interactions. The complex with α-2,8-disialyllactose identified a hydrogen bond between the N-acetyl group of the second sialic acid and the side chain of Ser195 (Figure 4B). However, the results from mutagenesis experiments demonstrate that a Ser195A mutation has no effect on either HA capacity or viral growth. Therefore, the observed contact is unlikely to have physiologic relevance. The interactions between σ1 and α-2,6-sialyllactose identified two hydrogen bonds that link the galactose to the protein and may help to stabilize the folded-back conformation of the ligand (Figure 4A). As both contacts involve main chain atoms of σ1, their functional significance cannot be easily probed by site-directed mutagenesis. Nevertheless, it is likely that the observed contacts lead to a modest increase in the affinity of σ1 for compounds terminating in α-2,6-linked sialic acid. It is unclear if such an increase is biologically significant.

Naturally occurring sequence variability at three amino acid positions (residues 198, 202, and 204) has been linked to the sialic acid-binding capacity of T3 σ1 [26], [42]. Our structures readily identify two of these residues, Arg202 and Pro204, as key determinants of sialic acid binding. The side chain of Arg202 forms a salt bridge with the sialic acid carboxylate group, while the Pro204 side chain stacks against the Arg202 guanidinium group. Moreover, the carbonyl oxygen in the peptide bond linking Leu203 and Pro204 forms a hydrogen bond with the sialic acid. Substitutions of either Arg202 or Pro204, as seen in the R202W and P204L variants, would decrease the affinity for sialic acid, and this is confirmed by the mutagenesis data. In contrast, the critical role of residue 198 in ligand recognition is not apparent from the crystal structures. Our mutagenesis data (Figure 5 and Figure 6), in conjunction with previous results [42], clearly demonstrate that Asn198 is required for successful sialic acid-dependent infection, with viruses carrying an N198D mutation having substantially reduced infectivity in MEL cells. However, the crystal structures show that Asn198 is not involved in direct or water-mediated contacts to any of the three oligosaccharides. Furthermore, the Asn198 side chain is solvent-exposed, forming a single hydrogen bond with the Asn189 side chain. Mutation of Asn189 to alanine does not affect sialic acid binding (Figure 5 and Figure 6), suggesting that the observed Asn198-Asn189 hydrogen bond is not relevant for ligand recognition. It is possible that the introduction of a negatively charged side chain at position 198, as is the case with the N198D mutation, leads to long-range electrostatic effects or structural rearrangements that indirectly affect receptor binding. However, given the distance of Asn198 from the binding site and its surface-exposed location, this possibility appears remote. We think it more likely that Asn198 serves as a contact point with a part of the functionally relevant glycan, which has not been included in the structural analysis. Although our results define the interactions of σ1 with terminal sialic acid, the actual receptor may be a more complex sialylated glycan, perhaps carrying several branches. Such complex receptor structures, which can be attached to proteins or lipids, have recently been identified as the true ligands for several adenovirus and polyomavirus capsid proteins [16]–[18]. Therefore, Asn198 may well define a second receptor contact point for reovirus σ1.

A large collection of structures of viruses or viral attachment proteins in complex with sialylated oligosaccharide receptors is available, and these have produced significant insights into mechanisms of sialic acid binding, receptor specificity, and viral pathogenesis [1]–[3], [5], [9], [11], [14], [16]–[18], [49]–[52]. However, the interactions observed between T3D σ1 and sialic acid differ in important ways from those found in all other virus-receptor complexes, offering new insights into the parameters that guide viral attachment and specificity. In all cases in which structures are available, the receptors are bound by a globular domain in a region that projects farthest from the viral capsid and is easily accessible for interactions with the cell surface. In contrast, the highly elongated T3D σ1 protein engages its carbohydrate ligand at its midpoint, about 150 Å away from the region that projects farthest from the virion. Although the σ1 protein possesses some flexibility at defined regions [19], [22], the location of the sialic acid-binding site would not appear optimal for engagement of membrane-bound receptors that feature sialylated ligands close to the membrane. The region of JAM-A that is engaged by the σ1 head domain is fairly close to the membrane [36]. Even when allowing for considerable flexibility between the σ1 head and body, it is difficult to envision a conformation in which the tail of σ1 is still inserted into the virus and the sialic acid binding site can closely approach the membrane. However, σ1 could more easily engage sialic acid that projects far above the membrane, perhaps by being located on a large protein or projecting from prominent loops.

Prior to this study, structural information had been available only for the C-terminal portion of the σ1 protein [22]. Based on analysis of that structure, as well as sequence comparisons with the related adenovirus fiber protein, full-length σ1 was predicted to fold into three distinct regions: an N-terminal α-helical coiled coil (termed the tail), a region containing eight consecutive β-spiral repeats (the body), and a globular β-barrel (the head). Our structural analysis of a fragment comprising the body and head domains show that this model must be revised, as we find an insertion of a short α-helical coiled coil that interrupts the β-spiral sequence in the body, replacing one β-spiral repeat with a helical structure. Thus, it is clear that the structure of σ1 features several transitions between α-helical and β-spiral regions. This topological relationship differs from that of the adenovirus fiber, in which the shaft domain is thought to consist entirely of β-spiral repeats [39]. Examination of the T3D body domain sequence shows that it contains a nearly perfect heptad repeat pattern, which is typical for α-helical coiled coils, in a short stretch of 14 residues (Figure 2). A similar pattern is observed in the T1L and T2J σ1 sequences, but a proline residue within the consensus makes it unlikely that these proteins also feature a continous α-helical coiled coil at the equivalent location.

**Figure 7:**
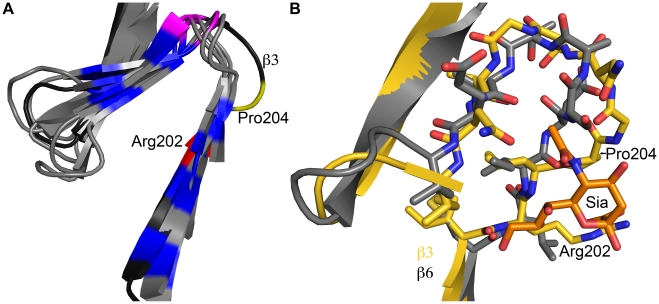
Structural adaption of the binding site. (**A**) Superposition of all seven β-spiral repeats. Repeat β3, which is shown in darker shading, interacts with sialic acid and deviates markedly in its structure from the other repeats. Conserved hydrophobic residues are colored in blue, the position in the β-turn that is usually occupied by proline or glycine is shown in magenta. Arg202 and Pro204, which are part of repeat β3, are highlighted in red and yellow, respectively. (**B**) Superposition of a prototypical β-spiral repeat (β6) onto the sialic acid binding repeat β3. Amino acids are shown in ball and stick representation, and residues Arg202 and Pro204 in β3 are labeled. Panel B is enlarged to provide a clearer view.

To our knowledge, the structures presented here are the first examples of any fibrous viral protein engaging a ligand via its repetitive fiber region. Other viral attachment proteins contain fibrous- or stalk-like structures, but they usually engage receptors with globular head domains placed on top of these structural elements, as observed in complexes of adenovirus fiber proteins with their receptors [7], [15], [18]. Globular head domains offer higher variability in engaging ligands and can more easily create recessed binding pockets suitable for high-affinity binding. Instead, fiber-like structures generally feature short connections between their repeating units and a relatively flat surface, limiting binding options. However, inspection of the β-spirals in σ1 reveals subtle modifications in a single repeat that allow it to create a shallow binding site for sialic acid. One of the hallmarks of β-spirals is a highly conserved β-turn between two strands, involving residues at positions g, h, i, and j (Figure 2). The residue at position j is usually a proline or glycine. This turn is enlarged by two amino acids in the σ1 repeat that engages sialic acid, transforming the turn into a small loop (Figure 7). Interestingly, Pro204 introduces a kink after a β-strand, causing the chain to deviate from the β-spiral motif at this position to provide a pocket for the ligand. Thus, alteration of the typical repeating motif identifies a ligand-binding site in the case of σ1. It is conceivable that similar aberrations in other fibrous protein sequences might also indicate binding sites. The location of a sialic acid binding site in an elongated fiber-like structure also raises the possibility of creating a small sialic acid binding cassette that could be transferred into a variety of trimeric fiber-like proteins constructed from α-helical coiled coils or β-spirals. Our work thus enhances an understanding of reovirus-glycan interactions and may also guide the construction of new sialic acid binding platforms to facilitate structure-function analyses and sialic acid-mediated cell targeting.

## Materials and Methods

### Protein Expression and Purification

The expression of soluble and properly folded T3D σ1 trimers was facilitated by appending a trypsin-cleavable trimerization domain based on the GCN4 leucine zipper [41] N-terminally to a cDNA encoding the entire σ1 body and head domains (amino acids 170–455). The construct was cloned into the pQE-80L expression vector, which encodes a non-cleavable N-terminal hexahistidine-tag. The protein was expressed in *E. coli Rosetta 2 DE3* (Novagen) at 20°C for 16 h post-induction or by autoinduction at 20°C for 48–72 h. Bacteria were lysed by two passages through an EmulsiFlex (Avestin) homogenizer and purified by Ni-IMAC using His-Trap-FF columns (GE-Healthcare). The immobilized protein was eluted by on-column digestion with 0.1 mg/ml trypsin at a flowrate of 0.1 ml/min for 12 h. Size-exclusion chromatography (Superdex-200, GE-Healthcare) was used as the final purification step.

### X-ray Structure Determination

Crystals were grown using 15% PEG200, 0.1 M MES (pH 6.5) as a precipitant. The crystals belong to space group P2_1_2_1_2 and contain one trimer in the asymmetric unit. Complexes with carbohydrate ligands were prepared by soaking crystals with the respective carbohydrate prior to data collection. The crystals were transferred into mother liquor supplemented with 10 mM carbohydrate, incubated for 5 min, and cryoprotected by incubation for 15 s in 35% PEG200, 0.1 M MES, 10 mM carbohydrate (pH 6.5).

Diffraction data were collected at the beamlines PXI (SLS) and ID14-4 (ESRF). Diffraction data were integrated and scaled using XDS [53], and the structure was solved by molecular replacement with AMoRe [54] using the structure of the T3D σ1 head (PDB ID 1KKE) as a search model. Refinement was performed with Refmac5 [55] and Phenix [56], and model building was done in Coot [57]. Ligands were fitted into weighted F_o_-F_c_ difference density maps at a contour level of 3σ and refined using the CCP4 library and user-defined restraints. Coordinates and structure factors for all three complexes have been deposited in the PDB data bank (www.rcsb.org) with accession codes 3S6X (complex with α-2,3-sialyllactose), 3S6Y (complex with α-2,6-sialyllactose) and 3S6Z (complex with α-2,8-di-sialyllactose).

### Cells

L929 cells [58] were maintained in Joklik's minimum essential medium (Sigma-Aldrich) supplemented to contain 5% fetal bovine serum, 2 mM L-glutamine, 100 U/ml of penicillin, 100 µg/ml of streptomycin, and 25 ng/ml of amphotericin B. MEL cells, previously designated T3cl.2 cells [59], were maintained in Ham's F-12 medium (CellGro) supplemented to contain 10% fetal bovine serum, 2 mM L-glutamine, 100 U/ml penicillin, 100 µg/ml streptomycin, and 25 ng/ml amphotericin B.

### Viruses

Recombinant reoviruses were generated by plasmid-based reverse genetics [43], [44]. Reovirus strains rsT3D (wild type), rsT3D-σ1N198D, rsT3D-σ1R202W, and rsT3D-σ1P204L were recovered using monolayers of L929 cells at approximately 90% confluence (3×10^6^ cells) in 60-mm dishes (Costar) infected with rDIs-T7pol [60] at an MOI of ∼0.5 TCID_50_. At 1 h post-infection, cells were co-transfected with ten plasmid constructs representing the cloned T3D genome using 3 µl of TransIT-LT1 transfection reagent (Mirus) per µg of plasmid DNA [43]. Reovirus strains rsT3D-σ1N189A, rsT3D-σ1S195A, rsT3D-σ1R202A, rsT3D-σ1L203A, rsT3D-σ1P204A, rsT3D-σ1G205A, and rsT3D-σ1N210A were recovered using BHK-T7 cells at 90% confluence (approximately 3×10^6^ cells) seeded in 60-mm dishes. Cells were co-transfected with five plasmids representing the cloned T3D genome using 3 µl of TransIT-LT1 transfection reagent (Mirus) per µg of plasmid DNA [44]. The amount of each plasmid used for transfection was identical to that described for L929 cell transfections. Following 3 to 5 days of incubation, recombinant viruses were isolated from transfected cells by plaque purification using monolayers of L929 cells [61]. For the generation of σ1 mutant viruses, pT7-S1T3D [43] was altered by QuikChange (Stratagene) site-directed mutagenesis. To confirm sequences of the mutant viruses, viral RNA was extracted from purified virions and subjected to Onestep RT-PCR (Qiagen) using *L1*- or *S1*-specific primers. (Primer sequences are available from the corresponding authors upon request.) The purified PCR products were subjected to sequence analysis for the presence of the introduced mutation in the *S1* gene segment and the noncoding signature mutation in the *L1* gene segment [43].

Purified reovirus virions were prepared using second-passage L929-cell lysate stocks of twice plaque-purified reovirus as described [20]. Viral particles were Freon-extracted from infected cell lysates, layered onto CsCl gradients, and centrifuged at 62,000 × *g* for 18 h. Bands corresponding to virions (1.36 g/cm^3^) [62] were collected and dialyzed in virion-storage buffer (150 mM NaCl, 15 mM MgCl_2_, 10 mM Tris-HCl pH 7.4). The concentration of reovirus virions in purified preparations was determined from an equivalence of one OD unit at 260 nm equals 2.1×10^12^ virions [62]. Viral titers were determined by plaque assay using L929 cells [61].

### HA Assay

Purified reovirus virions (10^11^ particles) were distributed into 96-well U-bottom microtiter plates (Costar) and serially diluted twofold in 0.05 ml of PBS. Calf erythrocytes (Colorado Serum Co.) were washed twice with PBS and resuspended at a concentration of 1% (vol/vol). Erythrocytes (0.05 ml) were added to wells containing virus particles and incubated at 4°C for at least 2 h. A partial or complete shield of erythrocytes on the well bottom was interpreted as a positive HA result; a smooth, round button of erythrocytes was interpreted as a negative result. HA titer is expressed as 10^11^ particles divided by the number of particles/HA unit. One HA unit equals the number of particles sufficient to produce HA. HA titers from three independent experiments were compared using an unpaired Student's *t* test as applied in Microsoft Excel. *P* values of less than 0.05 were considered statistically significant.

### Reovirus Infection of L929 and MEL Cells

L929 cells or MEL cells (2×10^5^ cells/well) were plated in 24-well plates (Costar) and incubated at 37°C for at least 2 h. Cells were adsorbed with reovirus strains at an MOI of 1 PFU/cell. Following incubation at room temperature for 1 h, cells were washed three times with PBS and incubated at 37°C for 24 or 48 h. Samples were frozen and thawed twice, and viral titers were determined by plaque assay [61]. For each experiment, samples were infected in triplicate. Mean values from three independent experiments were compared using an unpaired Student's *t* test as applied in Microsoft Excel. *P* values of less than 0.05 were considered statistically significant.
